# 

**Published:** 1869-07-15

**Authors:** 


### CHICAGO MEDICAL JOURNAL.

J. ADAMS ALLEN, M.D., LL.D., WALTER HAY, A.M., M.D., Editors.

All business letters for THE JOURNAL should be addressed to J. WOLCOTT HOOKER, Room 12, Union Building, Chicago, Ill.

All communications for publication should be addressed to DR. J. ADAMS ALLEN, 503 Michigan Avenue, Chicago, Ill.

TERMS—$3 PER ANNUM, IN ADVANCE.

$4 if not paid before 1st of July, in each year. Single Copies, 25 Cents.

